# Percutaneous fetoscopic spina bifida repair: effect on ambulation and need for postnatal cerebrospinal fluid diversion and bladder catheterization

**DOI:** 10.1002/uog.23658

**Published:** 2021-09-13

**Authors:** D. A. Lapa, R. H. Chmait, Y. Gielchinsky, M. Yamamoto, N. Persico, M. Santorum, M. M. Gil, L. Trigo, R. A. Quintero, K. H. Nicolaides

**Affiliations:** ^1^ Fetal Therapy Team Coordinator Hospital Infantil Sabara São Paulo Brazil; ^2^ Fetal Therapy Group Hospital Israelita Albert Einstein São Paulo Brazil; ^3^ Los Angeles Fetal Surgery, Department of Obstetrics and Gynecology Keck School of Medicine, University of Southern California Los Angeles CA USA; ^4^ Fetal Therapy, Helen Schneider Hospital for Women Rabin Medical Center Petah Tikva Israel; ^5^ Clinica Universidad Los Andes Santiago Chile; ^6^ Department of Clinical Sciences and Community Health University of Milan Milan Italy; ^7^ Fetal Medicine and Surgery Service, Fondazione IRCCS Ca' Granda, Ospedale Maggiore Policlinico Milan Italy; ^8^ Fetal Medicine Research Institute King's College Hospital London UK; ^9^ Department of Obstetrics and Gynecology Hospital Universitario de Torrejón Madrid Spain; ^10^ School of Medicine Universidad Francisco de Vitoria Madrid Spain; ^11^ BCNatal Fetal Medicine Research Center Barcelona Spain; ^12^ The Fetal Institute Miami FL USA

**Keywords:** artificial skin, biocellulose, cerebellar herniation, dura mater, fetoscopic surgery, fetoscopy, myelomeningocele, myeloschisis, prenatal therapy

## Abstract

**Objective:**

A trial comparing prenatal with postnatal open spina bifida (OSB) repair established that prenatal surgery was associated with better postnatal outcome. However, in the trial, fetal surgery was carried out through hysterotomy. Minimally invasive approaches are being developed to mitigate the risks of open maternal–fetal surgery. The objective of this study was to investigate the impact of a novel neurosurgical technique for percutaneous fetoscopic repair of fetal OSB, the skin‐over‐biocellulose for antenatal fetoscopic repair (SAFER) technique, on long‐term postnatal outcome.

**Methods:**

This study examined descriptive data for all patients undergoing fetoscopic OSB repair who had available 12‐ and 30‐month follow‐up data for assessment of need for cerebrospinal fluid (CSF) diversion and need for bladder catheterization and ambulation, respectively, from eight centers that perform prenatal OSB repair via percutaneous fetoscopy using a biocellulose patch between the neural placode and skin/myofascial flap, without suture of the dura mater (SAFER technique). Univariate and multivariate logistic regression analyses were used to examine the effect of different factors on need for CSF diversion at 12 months and ambulation and need for bladder catheterization at 30 months. Potential cofactors included gestational age at fetal surgery and delivery, preoperative ultrasound findings of anatomical level of the lesion, cerebral lateral ventricular diameter, lesion type and presence of bilateral talipes, as well as postnatal findings of CSF leakage at birth, motor level, presence of bilateral talipes and reversal of hindbrain herniation.

**Results:**

A total of 170 consecutive patients with fetal OSB were treated prenatally using the SAFER technique. Among these, 103 babies had follow‐up at 12 months of age and 59 had follow‐up at 30 months of age. At 12 months of age, 53.4% (55/103) of babies did not require ventriculoperitoneal shunt or third ventriculostomy. At 30 months of age, 54.2% (32/59) of children were ambulating independently and 61.0% (36/59) did not require chronic intermittent catheterization of the bladder. Multivariate logistic regression analysis demonstrated that significant prediction of need for CSF diversion was provided by lateral ventricular size and type of lesion (myeloschisis). Significant predictors of ambulatory status were prenatal bilateral talipes and anatomical and functional motor levels of the lesion. There were no significant predictors of need for bladder catheterization.

**Conclusion:**

Children who underwent prenatal OSB repair via the percutaneous fetoscopic SAFER technique achieved long‐term neurological outcomes similar to those reported in the literature after hysterotomy‐assisted OSB repair. © 2021 The Authors. Ultrasound in Obstetrics & Gynecology published by John Wiley & Sons Ltd on behalf of International Society of Ultrasound in Obstetrics and Gynecology.

## Abstract 2

This article's abstract has been translated into Spanish and Chinese. Follow the links from the abstract to view the translations.

Linked article: There is a comment on this article by Macedo Jr and Leal da Cruz. Click here to view the Correspondence.

## RESUMEN

**Reparación percutánea fetoscópica de la espina bífida: efecto sobre la deambulación y la necesidad de derivación de líquido cefalorraquídeo y cateterización vesical postnatales**

**Objetivo:**

En un ensayo para comparar la reparación prenatal con la postnatal de la espina bífida abierta (EBA) se estableció que la cirugía prenatal estaba asociada con un mejor resultado postnatal. Sin embargo, en dicho ensayo, la cirugía fetal se llevó a cabo mediante histerotomía. Se están desarrollando métodos mínimamente invasivos para mitigar los riesgos de la cirugía abierta materno‐fetal. El objetivo de este estudio fue investigar el impacto en el resultado postnatal a largo plazo de una técnica neuroquirúrgica novedosa para la reparación fetoscópica percutánea de la EBA fetal, como es la técnica de piel sobre biocelulosa para la reparación fetoscópica prenatal (SAFER, por sus siglas en inglés).

**Métodos:**

Este estudio analizó datos descriptivos de todos los pacientes sometidos a reparación fetoscópica de la EBA de los que se disponía de datos de seguimiento a los 12 y 30 meses para evaluar la necesidad de desviación del líquido cefalorraquídeo (LCR) y la necesidad de cateterización vesical y deambulación, respectivamente, los cuales procedían de ocho centros que realizan la reparación prenatal de la EBA mediante fetoscopia percutánea utilizando un parche de biocelulosa entre la placoda neural y el colgajo cutáneo/miofascial, sin sutura de la duramadre (técnica SAFER). Se utilizaron análisis de regresión logística univariantes y multivariantes para examinar el efecto de los distintos factores sobre la necesidad de derivación del LCR a los 12 meses y la deambulación y la necesidad de cateterización vesical a los 30 meses. Entre los posibles cofactores estaban la edad gestacional en el momento de la cirugía fetal y del parto, los resultados de ecografías preoperatorias del nivel anatómico de la lesión, el diámetro del ventrículo lateral cerebral, el tipo de lesión y la presencia de pie equinovaro, así como los hallazgos postnatales de pérdida de LCR en el momento del nacimiento, el nivel de motricidad, la presencia de pie equinovaro y la reversión de la hernia del rombencéfalo.

**Resultados:**

Se trató prenatalmente un total de 170 pacientes consecutivos con EBA fetal mediante la técnica SAFER. Se dio seguimiento a 103 de los bebés a los 12 meses de edad y a 59 a los 30 meses de edad. A los 12 meses de edad, el 53,4% (55/103) de los bebés no necesitaron una derivación ventriculoperitoneal o una tercera ventriculostomía. A los 30 meses de edad, el 54,2% (32/59) de los niños o niñas deambulaban de forma independiente y el 61,0% (36/59) no necesitaban cateterización vesical intermitente crónica. El análisis de regresión logística multivariante demostró que el tamaño del ventrículo lateral y el tipo de lesión (mielosquisis) proporcionaban una predicción significativa de la necesidad de desviación del LCR. Los parámetros indicativos significativos del estado deambulatorio fueron el pie equinovaro prenatal y los niveles motrices anatómicos y funcionales de la lesión. No hubo parámetros indicativos significativos de la necesidad de cateterización vesical.

**Conclusión:**

Los niños que se sometieron a una reparación prenatal de la EBA mediante la técnica percutánea fetoscópica SAFER obtuvieron resultados neurológicos a largo plazo similares a los descritos en la literatura tras una reparación de la EBA mediante histerotomía. © 2021 Los autores. Ultrasound in Obstetrics & Gynecology, publicada por John Wiley & Sons Ltd en nombre de la Sociedad Internacional de Ecografía en Obstetricia y Ginecología.

## 摘要

**经皮穿刺胎儿镜脊柱裂修复术:对步行的影响以及产后脑脊液分流和膀胱导尿的需要**

**目的:**

一项对比产前和产后开放性脊柱裂(OSB)修复术的试验，证实了产前手术与更好的产后预后有关。但在试验中，胎儿手术是通过剖宫产进行的。正在研制微创方式来缓解开放性产妇‐胎儿手术的风险。本研究目的在于调查一种用于胎儿OSB的经皮穿刺胎儿镜修复的新型神经外科技术(皮肤覆盖生物纤维的产前胎儿镜修复技术(SAFER))对长期产后预后的影响。

**方法:**

本研究调查了所有进行过胎儿镜OSB修复术的患者的描述性数据，这些患者有12至30个月的随访数据可用于分别评估脑脊液(CSF)分流和膀胱导尿的需要以及对步行的影响。这些患者所来自的八个中心进行了经皮穿刺胎儿镜产前OSB修复术，即在神经基板和皮肤/肌筋膜瓣之间使用一片生物纤维，而无需缝合硬脑膜(即SAFER技术)。使用了单因素和多元逻辑斯蒂回归分析法，针对第12个月CSF分流的需要、步行和在第30个月膀胱导尿的需要，研究了各种不同因素的影响。潜在辅因子有胎儿手术时及分娩时的胎龄、损害在结构上术前超声检查的结果、侧脑室内径、损害类型和双侧畸形足，以及出生时CSF渗漏的产后调查结果、行动水平、双侧畸形足的存在和后脑疝逆转。

**结果:**

总共连续有170名有胎儿OSB的患者，在产前以SAFER技术进行了治疗。在这些患者中，有103个婴儿在12个月大时进行了随访，还有59个婴儿在30个月大时进行了随访。在12个月大时，53.4%(55/103)的婴儿无需脑室腹膜分流术或第三脑室底造瘘术。在30个月大时，54.2%(32/59)的儿童能独立步行，而且61.0%(36/59)无需长期间歇性膀胱导尿。多元逻辑斯蒂回归分析法证实，对CSF分流需要的预测主要是由侧脑室大小及损害类型(脊髓裂)提供的。步行状态的主要预测因素是产前双侧畸形足以及损害在结构和功能上的运动水平。对于膀胱导尿则无重大预测因素。

**结论:**

采用经皮穿刺胎儿镜SAFER技术进行产前脊柱裂修复术的儿童，所获得的长期神经学预后类似于文献中所报告的经剖宫产辅助进行OSB修复术的预后。©2020作者。John Wiley&Sons Ltd代表国际妇产科超声学会发表在《妇产科超声》上。

**CONTRIBUTION**

*What are the novel findings of this work?*

The majority of children who had undergone percutaneous fetoscopic open spina bifida repair did not require a ventriculoperitoneal shunt or third ventriculostomy within the first 12 months of age (53%), were ambulating independently at 30 months of age (54%) and did not require chronic intermittent catheterization of the bladder at 30 months of age (61%).

*What are the clinical implications of this work?*

Long‐term neurological outcomes after percutaneous fetoscopic open spina bifida repair are similar to those after hysterotomy‐assisted repair.

## INTRODUCTION

The Management of Myelomeningocele Study (MOMS) reported that prenatal repair of open spina bifida (OSB) through maternal hysterotomy, compared to postnatal repair, was associated with a 50% reduction in the ventricular shunt rate (42% *vs* 80%) by the age of 12 months and a 50% increase in the rate of ambulation without the need for orthotics (42% *vs* 21%) by 30 months^1^, but there was no improvement in neurogenic bladder outcome^2^. However, such prenatal surgery necessitates deep general anesthesia using high‐dose inhaled agents to maintain intraoperative uterine relaxation^3^ and is associated with increased maternal and fetal risks for subsequent pregnancies. This includes a 10% risk of uterine rupture in the preterm period, between 26 and 32 weeks' gestation, with subsequent fetal demise occurring in two in five cases^4^, ^5^.

A percutaneous fetoscopic approach to prenatal OSB repair provides a potentially safer method than the open approach. Such a shift to minimally invasive treatment of the fetus has been a prime objective in the field of fetal therapy^6^, ^7^, ^8^. Feasibility of the fetoscopic approach for prenatal OSB repair was demonstrated in a series of animal studies^9^, ^10^, ^11^. In surgically created OSB in animal models, a biocellulose patch overlay not only protected the neural placode from the potentially caustic amniotic fluid, but also served as a scaffold to promote the development of a ‘neodura‐mater‐like tissue’ beneath the biocellulose layer^12^, ^13^. Thus, fetal healing properties were leveraged to create, in a matter of a few days, watertight coverage of the neural placode, thereby preventing further cerebrospinal fluid (CSF) leakage and leading to reversal of hindbrain herniation^8^, ^14^, ^15^. Interestingly, the less invasive approach of the skin‐over‐biocellulose for antenatal fetoscopic repair (SAFER) technique in sheep resulted in a more histologically normal appearing spinal cord compared to the classical neurosurgical approach used in the MOMS trial^16^. Preliminary data in our previously published studies in humans seem to corroborate these findings^8^, ^17^, ^18^.

The aim of this study was to report infancy and early childhood outcomes after percutaneous fetoscopic OSB repair using the SAFER neurosurgical approach, including need for CSF diversion at 12 months of age, and independent ambulation and need for chronic intermittent bladder catheterization at 30 months of age.

## PATIENTS AND METHODS

### Study population

Data from eight centers in six different countries that perform prenatal OSB repair using the percutaneous fetoscopic SAFER technique (in São Paulo (Brazil), Los Angeles (USA), Petah Tikva (Israel), Santiago (Chile), London (UK), Milan (Italy), Miami (USA) and New York (USA)) were examined. Of note, use of the biocellulose patch was not approved in Miami, Los Angeles and New York, so a dural substitute was instead used in these centers. First, we used data from children at 12 months of age or older to determine the need for CSF diversion by 12 months of age; CSF diversion was defined as either insertion of a ventriculoperitoneal shunt or endoscopic third ventriculostomy. Second, we used data from children at 30 months of age or older to determine the rate of and risk factors for the ability to ambulate without the need for an assistive device and the need for chronic intermittent bladder catheterization. Information on ambulation and need for chronic intermittent catheterization of the bladder was obtained from the attending physician, medical records and/or parents. The study (NCT04356703) was approved by the institutional review boards (IRB) of the Hospital Israelita Albert Einstein, São Paulo, Brazil (SGPP number: 2973‐17 on 1 April 2017), Keck School of Medicine, Los Angeles, CA, USA (IRB number: HS‐18‐00591 on 9 June 2018), Rabin Medical Center, Petah Tikva, Israel (IRB number: 0295‐19‐RMC on 21 May 2019), Clinica Universidad Los Andes, Santiago, Chile (ethical committee approval on 25 May 2017) and King's College Hospital, London, UK (New Clinical Procedures Committee approval on 25 January 2018).

### Prenatal diagnosis and assessment of spina bifida

The diagnosis of OSB with hindbrain herniation was established via preoperative ultrasound, and pre‐ and postoperative fetal magnetic resonance imaging was performed. Hindbrain herniation was defined as the cerebellar tonsils below the foramen magnum. Initially, the inclusion criteria were as follows: gestational age from 19 + 0 to 27 + 6 weeks; lesion level T1 to S1; hindbrain herniation; no other major anomaly; and normal karyotype. Exclusion criteria were: multiple gestation; previous uterine surgery; fetal kyphosis > 30°; cervical length < 2 cm; complete anterior placenta; low‐lying placenta; placental abruption; history of preterm birth; history of cervical incompetence; alloimmunization; positive maternal viral load for hepatitis and/or human immunodeficiency virus; large uterine fibroids; body mass index > 35 kg/m^2^; and maternal diabetes or hypertension. After completion of the CECAM (Cirurgia Endoscópica para Correção Antenatal da Meningomielocele) trial^8^, a few centers had some exclusion criteria removed, such as the gestational age and anatomical level requirements. Surgery was performed > 26 weeks' gestation due to late referral and/or to avoid extreme prematurity.

Lateral ventricular diameter was measured on ultrasound before surgery, and the larger of the two sides was used for the study. The anatomical level of the lesion was categorized as low if the lesion was at L5 or lower, intermediate if the lesion started at L3–L4 and high if the lesion was at or above L2. We now use motor level, rather than anatomical level, as an inclusion criterion^19^, ^20^. However, assessment of motor level was described after our study was started, and this evaluation was therefore not available for our initial cases; consequently, in this study, the anatomical level was used.

After discussion of all management options, patients who elected to proceed with fetoscopic treatment gave written informed consent and signed the IRB‐approved study consent forms. The technical issues of percutaneous fetoscopic OSB repair have been reported in detail previously^8^, ^18^, ^21^. Briefly, the neuroplacode is dissected and covered by the biocellulose patch, a myofascial flap is rotated and sutured to keep the patch in place, and then the skin is sutured in the midline or an artificial skin patch (Nevelia®, Symatese, Chaponost, France) is used. No relaxing incisions nor dura mater dissection or suture are performed.

### Delivery and neonatal management

Delivery was advised at 39 weeks' gestation, and Cesarean section was initially mandated based on the first version of the protocol. Vaginal delivery was allowed from Case 27 onwards. In cases of preterm prelabor rupture of membranes (PPROM) before 34 weeks' gestation, corticosteroids, intravenous ampicillin plus erythromycin for 2 days followed by oral amoxicillin plus erythromycin for 5 days, and a vaginal antimicrobial agent (miconazole and tinidazole) every 3 days was given until delivery. In cases of PPROM, elective delivery was recommended at 34 weeks' gestation. After birth, if a bilaminar patch was used, it was kept in place and protected using a Mepilex® wound dressing (Molnlycke, Goteborg, Sweden). Sutures were removed after spontaneous detachment of the silicone layer from the dermal matrix was noted. The dressing was changed at least every 2–3 days, until complete skin secondary intention healing was achieved. Daily assessments for CSF leakage from the surgically repaired site were performed while the neonate was admitted in the hospital. Leakage of CSF was defined as dripping from the lesion site and/or as a finding of persistent wet drapes. Postnatal motor level was assessed by a neurosurgeon and/or physiatrist at discharge and/or at outpatient visits, and the most recent findings were used. Functional motor level determined by ultrasound was divided into three categories: high (L1–L2), intermediate (L3–L4) and low (L5–S1).

### Outcome measures

Need for placement of a ventriculoperitoneal shunt or endoscopic third ventriculostomy was at the discretion of the attending pediatric neurosurgeon. Initially, we used the protocol established during the MOMS trial which was later modified in 2015^22^. Now the protocol is generally more restrictive, and at least one clinical sign (bulging fontanelle, split sutures or sunsetting sign) needs to be present for CSF diversion to be indicated. In cases in which CSF diversion was deemed necessary, the choice of shunt placement *vs* endoscopic third ventriculostomy was at the discretion of the attending pediatric neurosurgeon.

At birth, motor level was assigned from T12 to S2 using a more detailed clinical evaluation: muscle strength and tone; deep tendon reflexes; and coordination and movements. If the level was asymmetric, the ‘worse’ level was considered^23^. At 30 months, ambulation without the need for an assistive device was defined as no need for knee–foot orthoses, hip–knee–ankle–foot orthoses, trunk–hip–knee–ankle–foot orthoses (THKAFO), a cane, crutches or a walker^24^. Cases in which independent ambulation was not achieved were further subclassified as ambulators with assistive devices or non‐ambulators, i.e. those requiring a wheelchair.

Chronic intermittent bladder catheterization was defined as urethral clean catheterization for emptying the bladder every 3–4 h. The criteria for such treatment vary between centers. Some centers use a proactive approach for bladder catheterization, meaning that all spina bifida babies are initiated on chronic intermittent catheterization in the neonatal period. Only the Albert Einstein Hospital and the Milan center used a conservative approach^25^, in which chronic intermittent bladder catheterization is initiated only in cases with urinary tract ultrasound signs of neurogenic bladder, defined as increased bladder thickness, hydronephrosis, high residual volume, based on ultrasound examinations which are repeated every 3–6 months, or recurrent urinary infection.

The need to untether the spinal cord was based solely on the presence of clinical symptoms including loss of an acquired function (worsening or loss of gait), worsening of neurogenic bladder, local back pain and/or progressive scoliosis.

### Statistical analysis

Descriptive data were assessed for all patients undergoing fetoscopic OSB repair with available 12‐month follow‐up for assessment of CSF diversion and 30‐month follow‐up for assessment of bladder catheterization and ambulation. Comparisons between groups were performed using the Mann–Whitney *U*‐test for continuous variables and Fisher's exact test for categorical variables, with *post‐hoc* Bonferroni correction. Univariate and multivariate logistic regression analyses, with Firth's penalization when required, were used to determine which factors among prenatal and postnatal findings were significant predictors of outcome. The variance inflation factor (VIF), which represents the factor by which the variance is inflated, was used to assess multicollinearity; VIF values > 4 require further investigation, whereas VIF values > 10, which are a sign of serious multicollinearity, require correction. We used a non‐automated backwards elimination strategy to select variables included in the final model. All variables that were significant in the adjustment were included in the final model. The best model was selected using the Akaike information criterion. Significance was set at *P* < 0.05.

We conducted statistical analyses using package R software^26^. VIFs were calculated using the R package ‘jtools’^27^.

## RESULTS

### Study population

By 1 June 2020, 170 consecutive patients had undergone prenatal OSB repair using the SAFER technique in the eight different centers: 116 cases in São Paulo, 18 in Los Angeles, 10 in Petah Tikva, nine in Santiago, eight in London, five in Milan, three in Miami and one in New York.

In 54 of the 170 cases, fetal surgery was carried out less than 12 months prior to the time of writing. Of, the remaining 116, three cases were excluded because prenatal repair could not be completed; two of these were due to loss of access to the uterine cavity as a result of gas leaking to the maternal abdomen, and, in the third case, there was massive maternal gas embolism that prompted immediate delivery to ensure maternal safety. There was one fetal death, one pregnancy termination at the request of the parents, three prematurity‐related neonatal deaths and five additional deaths before the age of 12 months, of which three were infection‐related following insertion of a ventriculoperitoneal shunt. Thus, 103 cases were available for assessment of need for CSF diversion at 12 months of age: 87 cases from São Paulo, five from Petah Tikva, four from Milan, three from Santiago, two from Los Angeles, one from London and one from New York. A total of 60 cases should have been 30 months of age or older, but one died between 12 and 30 months, leaving 59 patients for assessment of independent ambulation and need for chronic bladder catheterization; all of these patients were treated *in utero* in Brazil.

### Outcome measures

Maternal and pregnancy characteristics of pregnancies assessed at 12 and 30 months after birth are summarized in Table 1. Table 2 summarizes the CSF diversion outcomes in 103 children at 12 months of age; 55 (53.4%) did not require a CSF diversion procedure, 42 (40.8%) had a ventriculoperitoneal shunt and six (5.8%) had endoscopic third ventriculostomy. Multivariate logistic regression analysis identified ventricular size and myeloschisis as prenatal risk factors for need of CSF diversion by 12 months of age (Table 3). The risk of CSF diversion within the first 12 months of age increased by 36% (odds ratio (OR), 1.36; 95% CI, 1.18–1.62) per 1‐mm increase in ventricular diameter above 10 mm and the risk was 4.91 times higher (OR, 4.91; 95% CI, 1.71–15.33) for cases with myeloschisis compared to those with myelomeningocele.

**Table 1:** Maternal and pregnancy characteristics of pregnancies with fetal open spina bifida repaired prenatally using percutaneous fetoscopy, assessed at 12 and 30 months after birth

Variable	12 months (*n* = 103)	30 months (*n* = 59)
Maternal age (years)	34.6 (31.1–38.2)	36.8 (32.4–39.4)
BMI (kg/m^2^)	26.3 (23.7–29.0)	25.8 (23.8–28.5)
Parity		
Nulliparous	57 (55.3)	33 (55.9)
Parous	46 (44.7)	26 (44.1)
Placental location		
Anterior	37 (35.9)	19 (32.2)
Posterior	52 (50.5)	27 (45.8)
Lateral	14 (13.6)	13 (22.0)
GA at fetoscopy (weeks)	27.0 (26.0–27.8)	26.7 (25.6–27.7)
Fetoscopy < 26 weeks	26 (25.2)	20 (33.9)
GA at birth (weeks)	32.8 (30.9–35.2)	32.7 (30.4–33.8)
Birth < 32 weeks	34 (33.0)	21 (35.6)
Prenatal ultrasound findings		
Anatomical level of lesion		
Low	56 (54.4)	34 (57.6)
Intermediate or high	47 (45.6)	25 (42.4)
Myeloschisis	25 (24.3)	16 (27.1)
Larger LV diameter (mm)	13.0 (10.0–15.5)	13.0 (11.0–16.0)
Larger LV diameter ≥ 15 mm	32 (31.1)	18 (30.5)
Bilateral talipes	16 (15.5)	10 (16.9)
Findings at birth		
Reversal of HH	84 (81.6)	54 (91.5)
CSF leakage	10 (9.7)	8 (13.6)
Bilateral talipes	19 (18.4)	9 (15.3)
Functional motor level		
Low	63 (61.2)	37 (62.7)
Intermediate or high	40 (38.8)	22 (37.3)
Postnatal complications		
CSF diversion	48 (46.6)	31 (52.5)
Bladder catheterization	—	23 (39.0)
Not walking independently	—	27 (45.8)

Data are given as median (interquartile range) or *n* (%).

BMI, body mass index; CSF, cerebrospinal fluid; GA, gestational age; HH, hindbrain herniation; LV, lateral ventricle.

**Table 2:** Characteristics of pregnancies with fetal open spina bifida repaired prenatally using percutaneous fetoscopy, according to whether cerebrospinal fluid (CSF) diversion was required within the first 12 months of age (*n* = 103) and need for bladder catheterization and ambulation within the first 30 months of age (*n* = 59)

					Ambulation		
	CSF diversion		Bladder catheterization				
Variable	Yes (*n* = 48)	No (*n* = 55)	Yes (*n* = 23)	No (*n* = 36)	Walking independently (*n* = 32)	Walking with assistance (*n* = 12)	Not walking (*n* = 15)
GA at fetoscopy (weeks)	27.3 (26.4–28.0)	26.6 (25.9–27.5)	26.7 (25.7–27.7)	26.9 (25.6–27.8)	26.4 (25.6–27.6)	26.4 (25.3–27.5)	27.6 (27.0–27.8)
Fetoscopy < 26 weeks	10 (20.8)	18 (32.7)	8 (34.8)	12 (33.3)	12 (37.5)	5 (41.7)	3 (20.0)
GA at birth (weeks)	32.7 (30.4–34.3)	33.1 (31.6–35.5)	32.7 (30.7–33.5)	32.9 (30.4–34.0)	32.5 (30.2–33.9)	32.2 (30.9–32.7)	33.4 (32.7–34.3)
Surgery–delivery interval (weeks)	5.75 (3.88–7.40)	6.75 (4.78–8.35)	5.80 (4.75–7.70)	5.90 (4.38–6.85)	5.7 (4.0–8.0)	5.95 (4.3–6.4)	5.90 (5.3–8.0)
Prenatal US findings							
Anatomical level of lesion							
Low	20 (41.7)	35 (63.6)*	14 (60.9)	20 (55.6)	25 (78.1)	4 (33.3)	5 (33.3)
Intermediate or high	28 (58.3)	20 (36.4)*	9 (39.1)	16 (44.4)	7 (21.9)	8 (66.7)*	10 (66.7)*
Myeloschisis	16 (33.3)	9 (16.4)	7 (30.4)	9 (25.0)	9 (28.1)	3 (25.0)	4 (26.7)
Larger LV diameter (mm)	14.3 (13.0–17.3)	11.0 (9.0–14.0)*	14.0 (11.0–17.0)	12.5 (10.0–15.0)	12.0 (10.0–14.0)	14.5 (10.8–18.0)	13.0 (11.0–16.8)
Larger LV diameter ≥ 15 mm	22 (45.8)	10 (18.2)*	8 (34.8)	10 (27.8)	6 (18.8)	6 (50.0)	6 (40.0)
Bilateral talipes	8 (16.7)	8 (14.5)	3 (13.0)	7 (19.4)	1 (3.1)	2 (16.7)*	7 (46.7)*
Findings at birth							
Reversal of HH	40 (83.3)	44 (80.0)	21 (91.3)	33 (91.7)	30 (93.8)	12 (100)	12 (80.0)
CSF leakage	7 (14.6)	3 (5.5)	4 (17.4)	4 (11.1)	6 (18.8)	2 (16.7)	0 (0)
Bilateral talipes	6 (12.5)	13 (23.6)	2 (8.7)	7 (19.4)	2 (6.3)	3 (25.0)	4 (26.7)
Functional motor level							
Low	26 (54.2)	36 (65.5)	12 (52.2)	25 (69.4)	27 (84.4)	5 (41.7)*	5 (33.3)*
Intermediate or high	22 (45.8)	19 (34.5)	11 (47.8)	11 (30.6)	5 (15.6)	7 (58.3)*	10 (66.7)*

Data are given as median (interquartile range) or *n* (%).

Comparisons between groups were performed using Mann–Whitney *U*‐test for continuous variables and Fisher's exact test for categorical variables, with *post‐hoc* Bonferroni correction with adjusted *P*‐value of < 0.025.

* Significant difference (*P* < 0.025) compared with CSF‐diversion group or walking‐independently group.

GA, gestational age; HH, hindbrain herniation; LV, lateral ventricle; US, ultrasound.

**Table 3:** Univariate and multivariate logistic regression analyses demonstrating factors providing a significant contribution to the prediction of need for postnatal cerebrospinal fluid (CSF) diversion within the first 12 months of age (*n* = 103) and need for bladder catheterization and not being able to walk independently at 30 months of age (*n* = 59) in children with fetal open spina bifida repaired prenatally using percutaneous fetoscopy

	CSF diversion				Bladder catheterization*		Not able to walk independently			
	Univariate analysis		Multivariate analysis		Univariate analysis		Univariate analysis		Multivariate analysis	
Independent variable	OR (95% CI)	*P*	OR (95% CI)	*P*	OR (95% CI)	*P*	OR (95% CI)	*P*	OR (95% CI)	*P*
*Prenatal predictors*										
Intercept	—	—	0.01 (0.003–0.05)	< 0.001	—	—	—	—	0.31 (0.13–0.66)	0.004
GA at fetoscopy (in weeks)†	1.22 (0.91–1.67)	0.184	—	—	0.92 (0.61–1.40)	0.704	1.22 (0.82–1.87)	0.335	—	—
Fetoscopy < 26.0 weeks	0.54 (0.21–1.31)	0.179	—	—	1.07 (0.35–3.21)	0.909	0.70 (0.23–2.08)	0.525	—	—
Ultrasound findings										
Anatomical level of lesion										
Low	Reference	—	Reference	—	Reference	—	Reference	—	Reference	—
Intermediate or high	2.65 (1.20–5.99)	0.017	2.42 (0.98–6.11)	0.057	0.80 (0.27–2.32)	0.687	7.14 (2.34–24.19)	0.001	5.25 (1.59–18.78)	0.008
Myeloschisis	2.56 (1.02–6.72)	0.049	4.91 (1.71–15.33)	0.004	1.31 (0.40–4.22)	0.647	0.89 (0.27–2.84)	0.850	—	—
Larger LV diameter (in mm)‡	1.30 (1.14–1.52)	< 0.001	1.36 (1.18–1.62)	< 0.001	1.10 (0.96–1.26)	0.183	1.11 (0.97–1.28)	0.128	—	—
Larger LV diameter ≥ 15 mm	4.90 (1.91–13.85)	0.001	—	—	1.87 (0.58–6.09)	0.293	3.71 (1.14–13.64)	0.036	—	—
Bilateral talipes	1.37 (0.45–4.11)	0.572	—	—	0.62 (0.12–2.53)	0.525	15.50 (2.60–297.98)	0.012	9.52 (1.40–191.69)	0.048
*Postnatal predictors*										
Intercept	—	—	0.64 (0.36–1.09)	0.103	—	—	—	—	0.37 (0.17–0.74)	0.007
GA at birth (in weeks)†	0.93 (0.81–1.06)	0.301	—	—	1.04 (0.84–1.28)	0.714	1.03 (0.85–1.25)	0.746	—	—
Surgery–delivery interval (in weeks)†	0.89 (0.77–1.02)	0.097	—	—	1.02 (0.83–1.24)	0.867	0.99 (0.80–1.21)	0.888	—	—
Findings at birth										
Reversal of HH	1.21 (0.25–6.47)	0.809	—	—	1.91 (0.23–40.01)	0.586	0.80 (0.09–7.07)	0.830	—	—
CSF leakage	2.96 (0.77–14.39)	0.132	—	—	1.68 (0.36–7.89)	0.495	0.35 (0.05–1.67)	0.220	—	—
Bilateral talipes	0.51 (0.17–1.45)	0.219	0.28 (0.08–0.95)	0.048	0.39 (0.06–1.83)	0.275	5.25 (1.13–37.73)	0.052	—	—
Functional motor level										
Low	Reference	—	Reference	—	Reference	—	Reference	—	Reference	—
Intermediate or high	1.60 (0.73–3.58)	0.244	2.84 (1.12–7.70)	0.033	2.08 (0.71–6.27)	0.184	9.18 (2.84–34.47)	< 0.001	9.18 (2.84–34.47)	< 0.001

* Multivariate analysis did not identify any significant predictors of need for bladder catheterization at 30 months.

† Per 1‐week increase.

‡ Per 1‐mm increase above 10 mm.

GA, gestational age; HH, hindbrain herniation; LV, lateral ventricle; OR, odds ratio.

Need for bladder catheterization at 30 months of age is summarized in Table 2; 36 (61.0%) children did not require chronic intermittent catheterization and there were no significant differences between those needing and those not needing catheterization in prenatal lesion level nor postnatal functional motor level. No independent predictors of bladder catheterization were identified (Table 3).

Ambulatory outcome at 30 months of age is summarized in Table 2; 32 (54.2%) children could walk without needing an assistive device, 12 (20.3%) were ambulating with an assistive device and 15 (25.4%) were non‐ambulatory. The functional motor level at the 30‐month assessment was classified as low in 37 (62.7%) cases, intermediate in 16 (27.1%) and high in six (10.2%). Multivariate regression analysis demonstrated that significant prediction for ambulation was provided by prenatal findings of bilateral talipes and anatomical level of the lesion and postnatal functional motor level (Table 3). The risk of not being able to walk independently at 30 months of age was 5.25 times higher (OR, 5.25; 95% CI, 1.59–18.78) for cases with an intermediate or high anatomical lesion compared to those with a low lesion, 9.52 times higher (OR, 9.52; 95% CI, 1.40–191.69) for cases with compared to those without prenatal bilateral talipes and 9.18 times higher (OR, 9.18; 95% CI, 2.84–34.47) for cases with intermediate or high postnatal functional motor level compared to those with a low level. A prediction model based on these three parameters and gestational age at surgery had 76% accuracy.

## DISCUSSION

### Main findings

There are four main findings of this study of patients that underwent percutaneous fetoscopic OSB repair. First, at 12 months of age, 53.4% did not require ventriculoperitoneal shunt or third ventriculostomy. Second, at 30 months of age, 54.2% were ambulating independently and 61.0% did not require chronic intermittent catheterization of the bladder. Third, preoperative lateral ventricular diameter and presence of myeloschisis were prenatal predictors of the need of CSF diversion by 12 months of age. Fourth, significant prediction for ambulation at 30 months of age was provided by prenatal bilateral talipes and anatomical and functional motor level of the lesion. Gestational age at fetal surgery, before or after 26 weeks, and gestational age at birth were not predictive of the three outcome measures of this study, but a larger number of patients will need to be investigated before exclusion of such potential associations.

### Comparison with results of previous studies and interpretation of results

The criteria and techniques for fetal surgery, as well as the definition of outcome measures, vary between studies, and, therefore, the conclusions that can be drawn from comparison of different series are limited. Nevertheless, the long‐term outcomes after percutaneous fetoscopic OSB repair compared favorably to previously published outcomes after hysterotomy‐assisted repair^1^, including no need for CSF diversion in 53% *vs* 60% of cases, ambulation without orthotics in 54% *vs* 42% and no need for chronic intermittent bladder catheterization in 61% *vs* 62%^2^. A recent study from Diehl *et al*. also showed that the fetoscopic approach provides at least similar long‐term outcomes as compared to hysterotomy‐assisted fetal surgery, with 48% requiring CSF diversion and 46% with independent ambulation at 30 months of age^28^. Furthermore, the neurosurgical repair technique that was used in the MOMS trial needs to be re‐evaluated, since both our repair technique and the modified neurosurgical technique described by Heuer *et al*.^29^ have improved outcomes. By adding the myofascial flap, their group improved significantly the rate of complete reversal of hindbrain herniation (71%^22^ *vs* 36%^1^ in the MOMS prenatal group), and no inclusion‐cyst removal was needed in their latest 45 cases^30^. In our cohort, 62.6% of cases had complete reversal of hindbrain herniation, and, so far, there were no cases requiring surgery for removal of inclusion cysts.

The physiology of a fetus is quite different from that of a neonate; consequently, not all techniques used in postnatal repair can achieve the same performance when used *in utero*. For example, traditional diaphragmatic hernia repair failed *in utero* due to the kink of the umbilical vein that occurred after the intestines were returned to the abdominal cavity; based on this understanding that the fetus is different from the neonate, the novel approach of tracheal occlusion resulted in relatively improved outcome^31^. Similarly, the idea that prenatal correction of OSB should be conducted in a similar fashion to that of postnatal repair is a concept that should be critically examined.

A fundamental question that this study raises is why the minimally invasive SAFER approach provided apparently similar or better neurological outcome as compared to ‘classic’ neurosurgical closure, which is a three‐layer repair (dura, aponeurosis and skin). One explanation is that, in the prenatal setting, primary closure of the dura with a suture may be detrimental to the neural elements due to reduced perfusion and to scar formation, as shown in our prior animal studies^16^, ^32^. In contrast, these same animal studies found, in a head‐to‐head comparison, improved preservation of neuroanatomy in the biocellulose repair group, with no CSF leakage at birth^16^. It is possible that this finding may translate to less cord tethering, since the biocellulose itself separates the neodura mater from the skin/muscle. Cord tethering is a long‐term complication of neonatal repair of OSB, and occurs when the medulla remains adherent to the overlying tissue^33^, ^34^. Lengthening of the spine during childhood and adolescence results in overstretching of the medulla, causing back and local pain, increasing scoliosis, swallowing problems and loss of motor and bladder function^34^. The problem with surgical release of the cord tethering is that it does not guarantee that the functions which the child lost will be recovered, which is one reason why an ambulating child may stop walking. Although our numbers are still small and preliminary (the children in this cohort have yet to reach their growth spurt), we found a 4% rate of need for surgery due to cord tethering, which is half the rate reported in the MOMS trial at 30 months^1^. However, it may be too premature to suggest that primary closure of the dura is not needed for prenatal repair.

The main problem with the percutaneous fetoscopic OSB repair technique is the high rate of PPROM and low gestational age at delivery. However, recent alterations of the surgical technique (not reflected in this early cohort) have resulted in improved short‐term outcome. Preliminary data suggest that, for example, after we initiated humidification of the CO_2_ during fetoscopy, these short‐term outcomes significantly improved; mean gestational age at birth increased from 32.5 to 34.5 weeks, and the rate of PPROM decreased from 67% to 38%^17^.

The optimal timing of prenatal surgery for OSB remains unknown. The upper gestational age at surgery for the MOMS trial was determined by the available information at that time, indicating no additional effect on the need for shunt when surgery was performed after 26 weeks^35^. Postponement of prenatal repair beyond 26 weeks' gestation provides the potential benefit of decreasing the risk of extreme prematurity at the risk of ongoing neurological damage and progression of disease. Although our cohort is relatively small, our data do not seem to suggest that surgical intervention beyond 26 weeks' gestation provides suboptimal outcome.

### Strengths and limitations

The main strength of this study is that it represents the first description of long‐term outcome after this novel neurosurgical approach using the SAFER technique for percutaneous fetoscopic OSB repair. The main limitation is the relatively small number of cases with 30‐month follow‐up. Another important weakness is that comparison with findings from the MOMS trial^1^ is limited by the fact that, unlike the MOMS trial, which used a standardized approach for prospective collection of data, in our study, varied criteria by different physicians making decisions were used for the three outcome measures, with an inevitable risk of selection bias. The surgical technique remains in the early phase of development, and various technical and equipment modifications remain ongoing. For instance, we are now adding a myofascial flap over the biocellulose patch and we changed the type of artificial skin patch.

### Conclusions

The findings of this study suggest that children with OSB who had undergone prenatal repair using the percutaneous fetoscopic SAFER technique achieved neurological outcomes similar to, if not better than, those reported in the literature after hysterotomy‐assisted repair. Although a randomized trial may ultimately be needed to compare fetoscopic and open fetal surgery, this is unlikely to take place because both techniques are evolving and many fetoscopy centers do not offer an open fetal surgery approach and *vice versa*.

## Data Availability

Research data are not shared.
